# Aligning the American Health Information Management Association Entry-level Curricula Competencies and Career Map With Industry Job Postings: Cross-sectional Study

**DOI:** 10.2196/38004

**Published:** 2022-07-07

**Authors:** Susan H Fenton, David T Marc, Angela Kennedy, Debra Hamada, Robert Hoyt, Karima Lalani, Connie Renda, Rebecca B Reynolds

**Affiliations:** 1 School of Biomedical Informatics University of Texas Health Science Center at Houston Houston, TX United States; 2 Department of Health Informatics and Information Management The College of St Scholastica Duluth, MN United States; 3 Commission on Accreditation for Health Informatics and Information Management Education (CAHIIM) Chicago, IL United States; 4 Loma Linda University Loma Linda, CA United States; 5 Department Internal Medicine Virginia Commonwealth University Richmond, VA United States; 6 Department of Health Information Technology and Health Information Management San Diego Mesa College San Diego, CA United States; 7 Department of Health Informatics and Information Management University of Tennessee Health Science Center Memphis, TN United States

**Keywords:** health information management, health workforce, healthcare industry, natural language processing, medical education, professional education, job recruitment, job website, web scraping, data mining

## Abstract

**Background:**

The field of health information management (HIM) focuses on the protection and management of health information from a variety of sources. The American Health Information Management Association (AHIMA) Council for Excellence in Education (CEE) determines the needed skills and competencies for this field. AHIMA’s HIM curricula competencies are divided into several domains among the associate, undergraduate, and graduate levels. Moreover, AHIMA’s career map displays career paths for HIM professionals. What is not known is whether these competencies and the career map align with industry demands.

**Objective:**

The primary aim of this study is to analyze HIM job postings on a US national job recruiting website to determine whether the job postings align with recognized HIM domains, while the secondary aim is to evaluate the AHIMA career map to determine whether it aligns with the job postings.

**Methods:**

A national job recruitment website was mined electronically (web scraping) using the search term “health information management.” This cross-sectional inquiry evaluated job advertisements during a 2-week period in 2021. After the exclusion criteria, 691 job postings were analyzed. Data were evaluated with descriptive statistics and natural language processing (NLP). Soft cosine measures (SCM) were used to determine correlations between job postings and the AHIMA career map, curricular competencies, and curricular considerations. ANOVA was used to determine statistical significance.

**Results:**

Of all the job postings, 29% (140/691) were in the Southeast, followed by the Midwest (140/691, 20%), West (131/691,19%), Northeast (94/691, 14%), and Southwest (73/691, 11%). The educational levels requested were evenly distributed between high school diploma (219/691, 31.7%), associate degree (269/691, 38.6%), or bachelor’s degree (225/691, 32.5%). A master’s degree was requested in only 8% (52/691) of the postings, with 72% (42/58) preferring one and 28% (16/58) requiring one. A Registered Health Information Technologist (RHIT) credential was the most commonly requested (207/691, 29.9%) in job postings, followed by Registered Health Information Administrator (RHIA; 180/691, 26%) credential. SCM scores were significantly higher in the informatics category compared to the coding and revenue cycle *(P=.*006) and data analytics categories *(P<.*001) but not significantly different from the information governance category (*P*=.85). The coding and revenue cycle category had a significantly higher SCM score compared to the data analytics category *(P<*.001). Additionally, the information governance category was significantly higher than the data analytics category (*P*<.001). SCM scores were significantly different between each competency category, except there were no differences in the average SCM score between the information protection and revenue cycle management categories (*P*=.96) and the information protection and data structure, content, and information governance categories (*P*=.31).

**Conclusions:**

Industry job postings primarily sought degrees, with a master’s degree a distant fourth. NLP analysis of job postings suggested that the correlation between the informatics category and job postings was higher than that of the coding, revenue cycle, and data analytics categories.

## Introduction

The field of health information management (HIM) originated in 1928, when the American College of Surgeons identified a need to improve clinical documentation and established the Association of Record Librarians of North America. Now known as the American Health Information Management Association (AHIMA), the organization delineates HIM as “the practice of acquiring, analyzing, and protecting digital and traditional medical information vital to providing quality patient care” [1].

Health information management, sometimes called medical records, continues to evolve rapidly due to many driving forces. The widespread adoption of electronic health record (EHR) systems has helped catalyze this movement [2]. As reported by the Office of the National Coordinator for Health Information Technology (ONC), by 2017, 96% of nonfederal acute care hospitals in the United States used certified EHRs [3]. Other recent technologies impacting the HIM field include the increased use of application programming interfaces (APIs), machine learning, artificial intelligence, natural language processing (NLP), voice recognition, and the Internet of Medical Things (IoMT). Newer data standards such as HL7’s Fast Healthcare Interoperability Resources (FHIR) often result in more data exchange among patients and hospitals [4]. The ONC certified an FHIR API intended to expedite patients’ requests for EHR data that required more HIM expertise and training [5]. The United States anticipates transitioning to the International Classification of Diseases 11th Revision (ICD-11) in the next few years, which will require further HIM expertise and training [6]. An increasing secondary use of health care data necessitates an increase in data management, governance, and analytics, usually under the purview of HIM [7].

Like most industries today, the health care sector, including HIM, is experiencing unprecedented technological innovations that are causing many downstream changes in job definitions and required skill sets. This changing landscape demands a more technologically oriented and knowledgeable workforce. For example, there is a growing need for better data literacy and analytical skills [2]. Education in essential domains provides a foundational skill set, allowing HIM professionals to develop and grow professionally. In 2015, Gibson reported HIM graduates require skills and knowledge in record management, data quality, health information analysis, access, privacy, confidentiality, and information systems and technology [8]. A recent study noted a demand for ongoing health information technology (HIT) education, as well as a variety of EHR skills, a knowledge of operational medical terminology, and an ability to communicate with senior management [9].

According to the US Bureau of Labor Statistics (BLS), jobs in medical records and health information specialties are projected to grow 9% between 2020 and 2030 [10]. There is a need for more HIM workers who are “industry ready.” Establishing and incorporating HIM core competencies can help achieve this goal. The AHIMA Council for Excellence in Education is tasked with setting these core curricula competencies. AHIMA’s HIM curricula competencies are divided into 6 domains for associate, undergraduate, and graduate degree levels. Typically, the Bloom taxonomy level for competency increases with the degree level of education across the domains. The AHIMA HIM curricula domains include 6 domains: (1) Domain I: data structure, content, and information governance; (2) Domain II: information protection: access, use, disclosure, privacy, and security; (3) Domain III: informatics, analytics, and data use; (4) Domain IV: revenue cycle management; (5) Domain V: health law and compliance; and (6) Domain VI: organizational management and leadership [11].

The AHIMA interactive career map displays career paths for HIM professionals. The current version has 4 categories of HIM job-related positions: (1) coding and revenue cycle, (2) informatics, (3) data analytics, and (4) information governance [12]. Presently, no academic literature has been identified that evaluates the usefulness of such career maps. One study by Madlock-Brown et al [13] compared the AHIMA career map to job postings to identify gaps.

An important question that needs to be addressed is whether the current training for HIM professionals in this evolving field is congruent with industry demands. Previous workforce research reviewed the jobs of AHIMA members, evaluated the workforce projections of the BLS, and examined the top skills of AHIMA members [14]. This study was based on the AHIMA membership data and not from actual job postings. Another study by Marc et al [15] reviewed global job categories in health informatics and information management. In this study, we sought to answer the question of training compared with industry demands by mining Indeed, a popular online job recruitment website for data on current job postings in HIM [16]. Indeed was chosen as a platform for extracting job posting data due to the volume of HIM-related jobs found on the website. An evaluation of other job posting websites yielded a lower return than Indeed. Additionally, Indeed was used previously for evaluating HIM-related job postings [15].

The primary aim of this study is to assess the alignment of current AHIMA curricula domains and HIM job postings identified from Indeed, and the secondary aim is to evaluate how well the AHIMA career map aligns to current HIM job postings found on Indeed.

## Methods

### Collection and Cleaning of Job Postings

**Figure 1:**
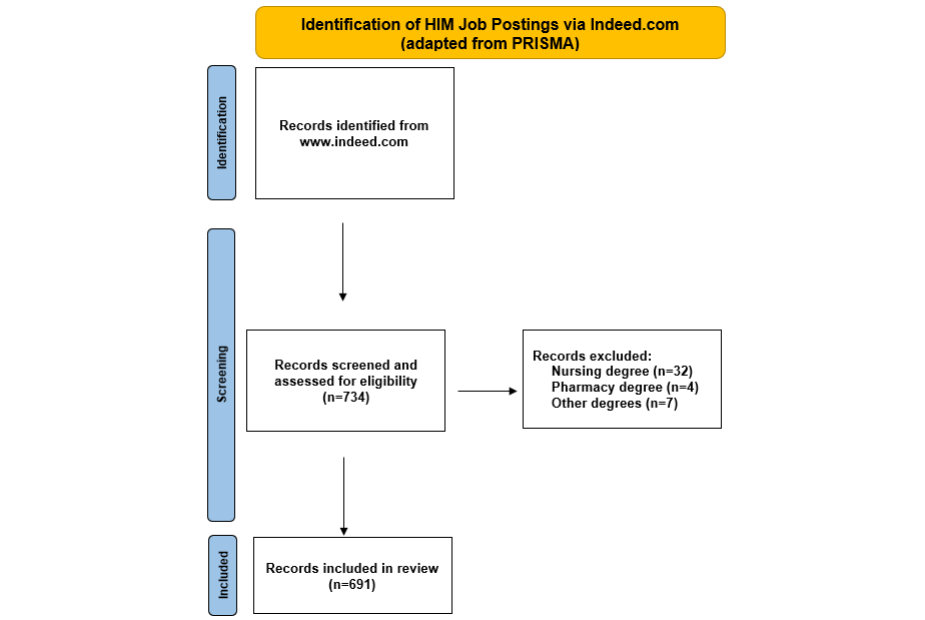
Identification of HIM job postings adapted from PRISMA (Preferred Reporting Items for Systematic Reviews and Meta-Analyses).

On June 18, 2021, job posting data were queried from Indeed using the keyword “health information management” (HIM) to extract the job title and any descriptive text from the job postings. The data were filtered to only full-time jobs posted within the last 14 days. A total of 734 job postings were returned from the query. The data were screened by expert review from 2 HIM management professionals for inclusion criteria. Subsequently, 43 job postings not relevant to HIM were removed, including 32 that required a nursing degree, 4 that required a pharmacy degree, and 7 that required other education not relevant to health information management. The remaining 691 job postings were included in the study. Figure 1 adapts the PRISMA (Preferred Reporting Items for Systematic Reviews and Meta-Analyses) flow diagram to display the inclusion and exclusion processes for job postings extracted from Indeed.

### Descriptive Statistics of Job Postings

The frequency and percentage of job postings were analyzed by geographic region, desired educational level, desired credentials, and length of time the positions were posted. Jobs were then categorized into geographical regions consistent with the categorization reported by the Commission on Accreditation for Health Informatics and Information Management Education (CAHIIM), the HIM accreditation body (Multimedia Appendix 1).

AHIMA curricular competencies for each educational level (associate, bachelor’s, and master’s) were organized by competency statement, domain, and Bloom taxonomy level for each competency statement. AHIMA career map positions were organized by domain, level, position, description, responsibilities, and skills required.

### Ethical Considerations

This study analyzed job postings and other publicly available job-related data. As such, it does not constitute human subjects research so no ethics approval was sought.

### Statistical Analysis of Job Postings

The team utilized natural language processing (NLP) to analyze the textual job posting data. The *requests* Python library was used to send an HTTP request to query Indeed for job postings using a URL to search for the keyword “health information management” and restrict the results to “full-time” and posted within the last “14 days.” The resulting HTML text was extracted to a CSV file using the *BeautifulSoup* Python library to extract specific <a> class text within the HTML files that corresponded to the job posting ID, title, company, location, day posted, URL to the posting, and job description.

The text in the resulting CSV file was preprocessed using the *re* Python library by first tokenizing the data sources into individual words, removing common English stop words and aberrant characters, converting the text to lowercase, and performing a lemmatizing protocol. Lemmatization is the process of finding the base form of a word to create a connection between related words to establish grammatical and semantic relationships.

The *gensim* Python library [17,18] was used to compute a word embedding similarity matrix by computing the cosine similarities between word embedding and retrieving the most similar terms for a given term using the pretrained Global Vectors for Word Representation (GloVe) embedding “glove-wiki-gigaword-50” [19]. A sparse term similarity matrix was generated that mapped terms and the indices of rows and columns based on the dictionary of all the text documents (job postings, AHIMA career map, and curricular competency categories) and the embedding similarity matrix. When creating the sparse term similarity matrix, a term frequency inverse document frequency (TFIDF) was used to specify the relative importance of the terms in the dictionary whereby the columns of the term similarity matrix were built in a decreasing order of importance of terms.

SCM was used as a measure of similarity between 2 documents. First described by Sidorov et al [20] in 2014, SCM utilizes a standard bag-of-words vector space model method but includes an evaluation of term similarity. SCM offers an evaluation of similarity between 2 documents even when they have no words in common, but the meaning of the words is the same. That is, SCM was used to measure the similarities between the text for each job posting and the text for each of the 4 AHIMA career map category and the text for each job posting and the text for each of the 6 AHIMA curricular competency categories.

The SCM score ranges from 0 to 1. The closer the value is to 1, the more similar the job posting documents are to a category. All 691 job postings received a SCM score for each of the 4 AHIMA career map and 6 curricular competency categories. A matrix of all SCM scores was generated to obtain statistics regarding the similarities of the job postings to the AHIMA career map and curricular categories.

Using the R Statistical programming language (R Core Team), SCM scores were summarized based on each AHIMA career map and curricular competency category [21]. Additionally, one-way ANOVA, which does not assume equal variance, was utilized to determine whether the SCM scores were significantly different among the AHIMA career map and curricular competency categories. Tukey HSD was used as a post hoc analysis to test for pairwise comparisons of SCM scores among categories [22]. The Python IDE PyCharm version 2022.1.1, RStudio version 2022.02.0 with R version 4.1.0 was used for analysis.

## Results

### Statistical Analysis of Job Postings

Table 1 depicts the descriptive statistics when comparing the average soft cosine measure (SCM) scores for job postings by each AHIMA career map category. One-way ANOVA established that the average SCM scores by AHIMA career map category resulted in significance (*F*_3_=48.21, *P*<.001). The Tukey honestly significant difference (HSD) test showed that SCM scores were significantly higher in the coding and revenue cycle category compared to the informatics (*P*=.006; 95% CI 0.004-0.04) and data analytics (*P*<.001; 95% CI 0.058-0.092) categories but not significantly different from the information governance category (*P*=.07; CI 0.001 to 0.033). The informatics category had a significantly higher SCM score than the data analytics category (*P*<.001; 95% CI 0.036-0.070). Additionally, the information governance category was significantly higher than the data analytics category (*P*<.001; 95% CI 0.042-0.076). There were no statistically significant differences between the information governance and informatics categories (*P*=0.85; 95% CI 0.012-0.022). Together, these results indicate that job postings are most strongly related to the coding and revenue cycle category of the AHIMA career map, followed by information governance, informatics, and data analytics categories.

Table 1 includes the average SCM for each AHIMA career map category. The coding and revenue cycle category had the highest mean SCM score (0.53), followed by the information governance (0.50) and informatics (0.50) categories. The data analytics category had the lowest mean SCM score (0.44) compared to the other categories.

Table 2 depicts the descriptive statistics when comparing the average SCM scores for job postings by AHIMA competencies. One-way ANOVA established that the average SCM scores by AHIMA competencies resulted in significance (*F*_5_=82.72, *P<.*001). Tukey HSD test showed that SCM scores were significantly different between each competency category, except there were no differences in the average SCM score between Domain III and Domain VI *(P=*.29; 95% CI 0.004-0.029) and between Domain III and Domain IV (*P*=.513; 95% CI 0.007-0.027). More specifically, Domain I had a significantly lower average SCM score compared to Domain II (*P*<.001; 95% CI 0.095-0.128), Domain III (*P*<.001; 95% CI 0.050-0.084), Domain IV (*P*<.001; 95% CI 0.040-0.074), Domain V (*P*<.001; 95% CI 0.021-0.055), and Domain VI (*P*<.001; 95% CI 0.063-0.096). Domain V had a significantly lower average SCM score compared to Domain II (*P*<.001; 95% CI 0.057-0.090), Domain III (*P*<.001; 95% CI 0.012-0.046), Domain IV (*P*=.02; 95% CI 0.002-0.036), and Domain VI (*P*<.001; 95% CI 0.025-0.058). Domain IV had a significantly lower average SCM score compared to Domain II (*P*<.001; 95% CI 0.034-0.071) and Domain VI (*P*=.002; 95% CI 0.006-0.039). Finally, Domain II had a significantly higher average SCM score compared to Domain III (*P*<.001; 95% CI 0.028-0.061) and Domain VI (*P*<.001; 95% CI 0.015-0.049). These results indicate that the job postings are most strongly related to information protection competencies, followed by competencies in organizational management and leadership/informatics/analytics/data use, revenue cycle management, health law and compliance, and then the data structure/content/information governance categories.

Table 2 includes the average SCM for each domain. Domain II was the highest (0.43), followed by Domain VI (0.40), Domain III (0.39), Domain IV (0.38), Domain V (0.36), and Domain I (0.32).

While job titles were not isolated for analysis, we analyzed selected postings to gain further insight into their unique resulting attributes. Two job postings, Data Analyst IV and Clinical Systems Analyst, matched 50% or more of all 6 AHIMA curriculum domains. Both positions required a bachelor’s degree and articulated the need for applicants to work independently. The foci of the positions were on data management and information system development. Four positions did not match across the 6 AHIMA curriculum domains; by position title, these included Senior Medical Coder, Document Imaging Medical Records Specialist, Quality Data Analyst, and Medical Record Clerk. The top matching positions within each AHIMA curriculum domain included Domain 1: Manager of NCQA Accreditation and Health Informatics; Domain II: Clinical Informatics Educator; Domain III: Senior Health Informatics Analyst; Domain IV: Data Analyst; Domain V: Clinical Informatics Educator; and Domain VI: Clinical System Analyst. Qualitatively, the significant presence or absence of domain matching for these HIM postings reflects potential current HIM workforce trends.

**Table 1:** Summary of soft cosine measure (SCM) scores by the American Health Information Management Association (AHIMA) career map.

	Coding and revenue cycle	Informatics	Data analytics	Information governance
Minimum	0.04	0.03	0.03	0.01
First quartile	0.45	0.41	0.37	0.43
Median	0.53	0.51	0.44	0.52
Mean	0.52	0.50	0.44	0.50
Third quartile	0.61	0.59	0.52	0.59
Maximum	0.78	0.84	0.74	0.80

**Table 2:** Summary of SCM scores by AHIMA curricula domains.

	Domain I	Domain II	Domain III	Domain IV	Domain V	Domain VI
Minimum	0	0	0	0	0	0
First quartile	0.27	0.34	0.32	0.33	0.29	0.34
Median	0.33	0.44	0.39	0.39	0.37	0.41
Mean	0.32	0.43	0.39	0.38	0.36	0.40
Third quartile	0.39	0.53	0.46	0.45	0.44	0.48
Maximum	0.52	0.84	0.67	0.60	0.63	0.65

### Descriptive Statistics of Job Postings

Our data include the 691 jobs extracted from Indeed. The results of the geographic distribution, demonstrates that most of the jobs posted were in the Southeast (203/691, 29%), followed by the Midwest (140/691, 20%), West (131/691, 19%,), Northeast (94/691, 14%), and Southwest (73/691, 11%) regions. The “national” regional category was added to our analysis to represent jobs listed as entirely remote, meaning they could be accomplished anywhere in the United States and were not geographically categorized. National jobs represented 7% (50/691) of all job postings.

Within the 14-day time frame from when the job postings were collected, 19.7% (136/691) had been posted 2 days prior to the data collection, followed closely by those posted 1 day prior (100/691, 14.5%) and then posted on the same day (74/691, 10.7%). The percentage of jobs posted declined the further the day of the data collection was from the date of posting, with only 1.5% (10.4/691) of jobs posted 14 days prior to collection.

The educational level requested in the job postings was generally evenly distributed between high school diploma (219/691, 31.7%), associate degree (269/691, 38.6%), or bachelor’s degree (225/691, 32.5%). A master’s degree was requested in only 8% (58/691) of the postings, with 72% (42/58) listing the degree as preferred and 28% (16/58) as required. Finally, the frequency and percentage of job postings that listed specific credentials in the qualifications and whether they were required or preferred were reported. The most requested credential was Registered Health Information Technologist (RHIT) at 29.9% (207/691), followed closely by the Registered Health Information Administrator (RHIA) at 26.0% (180/691). Additionally, the Certified Coding Specialist (CCS) credential was included in 18.1% (125/691) of job postings, the Certified Professional in Healthcare Quality (CPHQ) credential was requested in 0.6% (4/691) of the postings, and the Certified Professional in Healthcare Information and Management Systems (CPHIMS) credential was only required in 0.3% (2/691) of the postings. We examined only the RHIT and RHIA credentials to determine whether they were required or preferred. When requesting either credentials, 14% (98/691) required one or the other, while 9% (66/691) preferred either the RHIT or RHIA. When focused on a single credential, the numbers were much smaller, with 3% (23/691) requiring the RHIT and 2% (20/691) listing the credential as preferred. Only 1% (7/691) of the postings required the RHIA and 1% (9/691 postings) listed the credential as preferred (Tables 3-5).

**Table 3:** Educational levels requested.

Degree required	Value, n (%)^a^
High school diploma	219 (31.7)
Associate degree	269 (38.9)
Bachelor’s degree	225 (32.5)
Master’s degree	58 (8.4)

^a^Percentage totals more than 100% as more than 1 educational requirement may be mentioned in the same job posting.

**Table 4:** Credentials.

Credential listed	Value, n (%)
RHIA^a^	180 (26.0)
RHIT^b^	207 (29.9)
CCS^c^	125 (18.1)
CPHIMS^d^	2 (0.5)
CPHQ^e^	4 (0.6)

^a^RHIA: Registered Health Information Administrator.

^b^RHIT: Registered Health Information Technologist.

^c^CCS: Certified Coding Specialist.

^d^CPHIMS: Certified Professional in Healthcare Information and Management Systems.

^e^CPHQ: Certified Professional in Healthcare Quality.

**Table 5:** Required versus preferred credentials

Credential	n, %
Either RHIA^a^/RHIT^b^ required	98 (14.2)
Either RHIA/RHIT preferred	66 (9.6)
RHIT required	23 (3.3)
RHIA required	7 (1.0)
RHIT preferred	20 (2.9)
RHIA preferred	9 (1.3)

^a^RHIA: Registered Health Information Administrator.

^b^RHIT: Registered Health Information Technologist.

## Discussion

### Principal Findings

Our study results indicate that most HIM jobs require an associate degree or above. Many require an RHIT or RHIA credential with more opportunities in the Southeast region and a number of jobs closely aligned with coding and revenue cycle related careers. This aligns with the study by Madlock-Brown et al [13], which compared the AHIMA career map to Simply Hired job postings in 2019 [13]. However, the Indeed job postings did not strongly associate with any of the AHIMA curricular competencies despite significant differences amongst the categories. Additionally, the results indicate a similarity to informatics. Given that informatics is often identified as a skill set leveraged across professions, it was not addressed in singularity but brings up a potential research question that will require focus and analysis in a future study.

AHIMA curricular competencies had significant differences among the categories. Potential causes include delays between the time of job analysis, development competencies by the AHIMA CEE, and additional time to implement revised competencies for accreditation. There may be an opportunity to have the CAHIIM educational programs provide feedback to AHIMA regarding the educational program implementation of curricular competencies.

A SCM score closer to 1 indicates stronger similarity between the job postings and the categories evaluated. The mean scores for the comparison of the job postings to the AHIMA career map ranged from 0.44 to 0.53, while the mean scores for the comparison of the job postings to the AHIMA curricular competencies ranged from 0.32 to 0.43. These SCM scores indicate that the job postings have a moderate similarity to the AHIMA career map and a moderate to low similarity to the AHIMA curricular competencies.

The moderate similarity of the job postings to the AHIMA career map categories reveals that coding and revenue cycle jobs are prominent in these professions. However, the career map does not appear to fully capture the range of jobs for which HIM professionals are potentially qualified [13]. This may be due to the fast pace of change in health care and variances in the jobs that HIM professionals hold.

Our study findings indicate a moderate to low similarity of job postings to the curricular competencies. This suggests that the qualifications and requirements listed in job postings do not closely align with the AHIMA curricular competencies. This misalignment can be attributed to a number of factors, including a variability in the terminology used in higher education and industry. Another consideration is the time delay for curriculum to be vetted and disseminated, which typically lags behind the fast pace of change in the industry and in job postings. Another factor is the range from entry-level positions in HIM to higher level positions resulting in varied job qualifications and inconsistent terminology.

### Strengths and Limitations

One of the strengths of this research is that it is the first of its kind that aims to determine the usability and accuracy of the AHIMA career map, as it applies to health information management job postings found on Indeed. This study analyzed HIM job postings for all 50 US states and the District of Columbia, using data from Indeed, providing a snapshot of results based upon CAHIIM Annual Program Analysis Report (APAR) regions. Another strength of this study is its cross-sectional design, which provides a snapshot of the prevalence of HIM jobs nationally during a 2-week period.

As with all research studies, this study has some limitations. First, this study analyzed job posts on one job posting website and was limited to a certain time frame. Additionally, its nonexperimental design precludes establishing cause and effect relationships. Specific sampling decisions driven by practicality and time constraints are acknowledged. Establishing the search criteria of “health information management” potentially excluded HIM domain positions that did not include this title, such as Assistant Vice President (VP) of Revenue Integrity and Information Management.

There are limitations to using the AHIMA career map for comparison, as these jobs represent only AHIMA members and do not include those in the workforce that are not AHIMA members. The emerging roles on the career map were developed by focus groups and do not represent AHIMA member data, and the career map was last updated in 2016. Further, frequencies of specific job titles are not included in the AHIMA career map. Madlock-Brown et al [13] studied the AHIMA career map and found that many of the job titles found therein were not in the Simply Hired job search. Another challenge of the AHIMA career map is the inclusion of jobs from entry-level to higher levels, so the job varies from entry-level Patient Registration Clerk to VP of Compliance.

### Areas of Future Research

Researchers can use our study results to expand the literature and gaps in knowledge in this area in HIM workforce studies. One area of future research is to expand the search criteria for job postings from “health information management” to other search terms, such as “health data analyst,” or to search skills required beyond the degree. Another area of future research is to analyze HIM job postings for all 50 US states and the District of Columbia based on US Census regions. Additionally, future research can also explore usage of other NLP techniques, such as sentiment analysis or keyword extraction.

With the wide range of positions in HIM, educators may find it useful to identify jobs requiring on-the-job training. Exploring HIM professional job roles and attempting to eliminate the clerical roles associated with some tasks may be more meaningful to educators and students who are exploring the skills needed for the future HIM workforce.

This study highlights an opportunity to further explore content-based accreditation to meet the needs of end users, such as employers. There is also a need to explore educational programs primarily via competencies as opposed to degree title.

### Conclusions

The HIM field is continually evolving. This study analyzed HIM job postings to examine alignment with HIM domains and found that industry job postings primarily sought educational qualifications at or below the bachelor’s degree level. This is inconsistent with the 2017 AHIMA whitepaper, HIM Reimagined, in which the CEE calls for the percentage of AHIMA members with graduate degrees to double from 10% to 20% by 2027 [23]. The NLP analysis suggested the correlation between informatics and job postings was higher compared to the revenue cycle, coding, and data analytics categories. These findings should be reviewed carefully by the AHIMA CEE to ensure the accreditation competency domains are congruent with jobs offered by employers. At the same time, educators need to engage fully with those who employ their graduates. The accreditation competency domains represent the baseline for educational programs. Therefore, HIM educators should incorporate additional content to meet the needs of employers. There is great diversity in the job titles, educational requirements, and skills for jobs when searching for HIM positions. This is both exciting and challenging, as jobs titles and skills will continue to change to meet workforce needs. As jobs and competencies continue to evolve in the big data, machine learning, and artificial intelligence era, professional associations such as AHIMA, accreditors such as CAHIIM, HIM educators, and industry leaders must collaborate to align the HIM workforce needs of the health care industry with educational programs.

## Appendix

Multimedia Appendix 1 Commission on Accreditation for Health Informatics and Information Management Education (CAHIIM) Annual Program Analysis Report (APAR) region assignments.

## Abbreviations

AHIMA: American Health Information Management Association
AI: artificial intelligence
APAR: Annual Program Analysis Report
API: application programming interface
BLS: US Bureau of Labor Statistics
CAHIIM: Commission on Accreditation for Health Informatics and Information Management Education
CEE: Council for Excellence in Education
CPHIMS: Certified Professional in Healthcare Information and Management Systems
CPHQ: Certified Professional in Healthcare Quality
EHR: electronic health records
FHIR: Fast Healthcare Interoperability Resources
GloVe: Global Vectors for Word Representation
HIM: health information management
HIT: health information technology
HSD: honestly significant difference
ICD-11: International Classification of Diseases 11th Revision
IoMT: Internet of Medical Things
ML: machine learning
NLP: natural language processing
ONC: Office of the National Coordinator for Health Information Technology
PRISMA: Preferred Reporting Items for Systematic Reviews and Meta-Analyses
RHIA: Registered Health Information Administrator
RHIT: Registered Health Information Technologist
SCM: soft cosine measures
TFIDF: term frequency inverse document frequency
VP: vice president
