# Supplementary figures and images for: Aligning the American Health Information Management Association Entry-level Curricula Competencies and Career Map With Industry Job Postings: Cross-sectional Study
Source: JMIR Med Educ. 2022 Jul 7;8(3):e38004. doi: 10.2196/38004 (PMC9305438; doi:10.2196/38004)

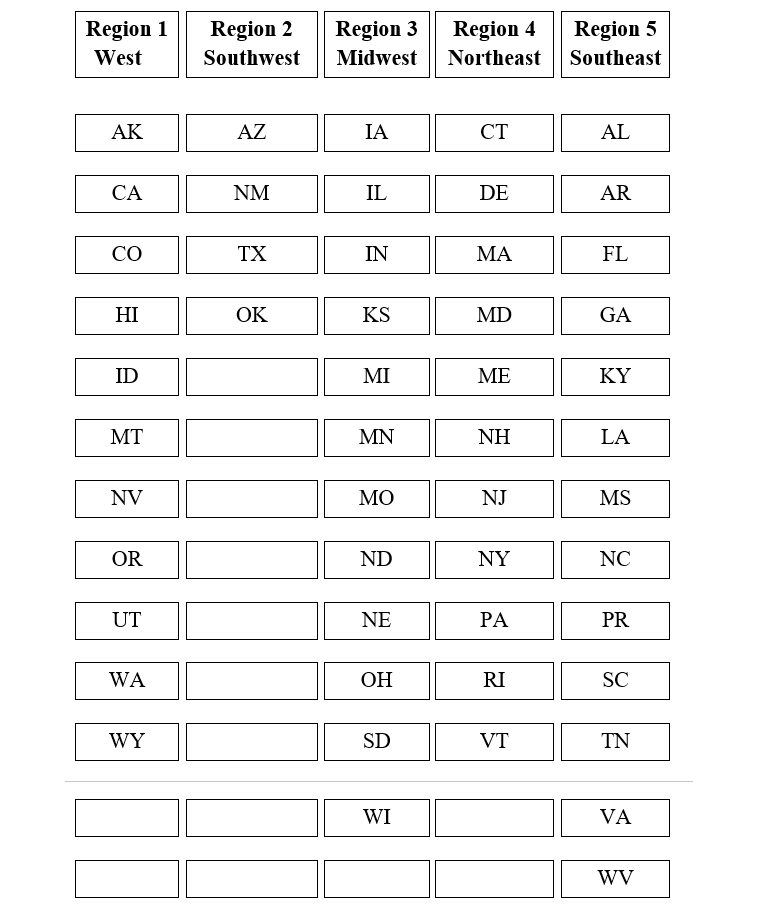

Supplement: Multimedia Appendix 1 [file mededu_v8i3e38004_app1.png]
